# Fabrication of Hybrid Silver Microstructures from Vermiculite Templates as SERS Substrates

**DOI:** 10.3390/nano10030481

**Published:** 2020-03-07

**Authors:** Nicolas Pazos-Perez, Luca Guerrini, Ramon A. Alvarez-Puebla

**Affiliations:** 1 Department of Physical and Inorganic Chemistry and EMaS, Universitat Rovira I Virgili, Carrer de Marcel∙lí Domingo s/n, Edifici N5, 43007 Tarragona, Spain; nicolas.pazos@urv.cat; 2 Institució Catalana de Recerca i Estudis Avançats (ICREA), Passeig Lluís Companys 23, 08010 Barcelona, Spain

**Keywords:** plasmonics, template-assisted synthesis, vermiculite, surface-enhanced Raman scattering, sensing

## Abstract

There is great interest in developing complex, 3D plasmonic materials with unusual structural properties. This can be achieved via template-assisted approaches exploiting scaffold elements to engineer unique plasmonic substrates, which would be otherwise impossible to synthesize. Herein, we present a novel, simple, and low-cost template-assisted method for producing interconnected 3-D silver microstructures by utilizing vermiculite, a well-known silicate, as both in-situ reductant and template for silver growth. The silicate network of the vermiculite can be easily removed by dissolution with hydrofluoric acid, which, simultaneously, leads to the formation of a magnesium fluoride skeleton supporting a plasmonically active silver film. Optical, morphological, and chemical properties of the materials were extensively investigated, revealing, for example, that hybrid silver microstructures can be exploited as valuable SERS substrates over a broad spectral range of excitation wavelengths.

## 1. Introduction

Template-assisted synthesis of nanostructured plasmonic materials is an extremely powerful approach for generating substrates with unique structures, morphologies, and properties [1,2]. In fact, exploiting pre-existence structures as a scaffold for nanostructural growth enables the fabrication of novel classes of materials, which are otherwise impossible to produce [3,4,5,6,7]. Among the many examples of structurally diverse templates, naturally occurring materials, including biological substances, have been exploited for this purpose using bottom-up approaches [8,9,10,11]. Typically, metal ions first coordinate functional groups on template-exposed surfaces and, subsequently, a reducing agent is added to yield the metallic structure [12]. Such an approach provides very simple and cost-effective routes for generating complex nanostructured materials.

Among the diverse classes of clay minerals exploited as natural absorbents, smectitic 2:1 clays such as vermiculite have demonstrated high efficiency at sequestering metal cations such as Ag^+^ and Cu^2+^ from aqueous solutions [13,14,15]. Vermiculites are hydrated magnesium–iron–aluminum silicates that typically exist as shiny flakes. Their porous structures comprise silicate layers organized in a 1 to 2 conformation (one aluminum octahedral sheet is sandwiched between two silicate tetrahedral ones). Such conformation leads to the formation of interlayer gaps, which mainly include cations to counterbalance the negative charge of the siloxamic cavities of vermiculite surfaces [16,17]. Electrostatically interacting metal ions at planar sites (outer-sphere complexes) can be efficiently exchanged. For instance, it was reported that silver ions exchange with Na^+^ ions [17] and, remarkably, can exist on the vermiculite surfaces both in the Ag^+^ and Ag^0^ forms [4,5]. Thus, vermiculite has the potential to be used as a low-cost template for the fabrication of complex silver substrates. 

In this work, we exploited the structural features and surface chemistry of vermiculites to be used simultaneously as in-situ reductants and scaffolds for the low-cost, straightforward, easily scalable synthesis of hybrid silver microstructures. Notably, the silicate network layer of vermiculite can be easily removed by dissolution with hydrofluoric acid, which leaves behind a backbone structure of magnesium fluoride (MgF_2_) that supports a plasmonically active 3-D silver construct. MgF_2_ is characterized by a high transmittance in the spectral range of interest for SERS (from ~100 nm to ~8 μm) and excellent mechanical resistance, [18] which make it a very intriguing material in the construction of hybrid plasmonic substrates [19]. Optical, chemical, and morphological properties of the hybrid Ag microstructures were extensively characterized and investigated, via UV-Vis spectroscopy, surface-enhanced Raman spectroscopy (SERS), environmental scanning electron microscopy (ESEM) coupled with energy-dispersive X-ray analysis (EDX), and transmission electron microscopy (TEM). Among various intriguing features, it is worth highlighting that hybrid silver microstructures displayed a high SERS activity across a wide spectral range of excitation wavelengths. 

## 2. Materials and Methods 

### 2.1. Materials 

All materials were of the highest purity available and obtained from Sigma-Aldrich (St. Louis, MO, US) and Fisher Scientific (Hampton, NH, US).

### 2.2. Sample Preparation 

0.26 g of vermiculite were immersed into 40 mL of a 2 M NaCl solution and left shaking overnight. This step was repeated three times, followed by three washing cycles with 40 mL of Milli-Q water. The resulting vermiculite was then immersed into 10 mL of a 0.1 M solution of AgNO_3_ and left shaking overnight. This step was repeated three times, followed by three washing cycles with Milli-Q water to remove the excess of unreacted silver ions. Finally, replacement of the silicate networks with magnesium fluoride (MgF_2_) backbone was achieved upon ca. 3 h immersion into a hydrofluoric acid solution (30%) in a polypropylene tube. The brown-grey sediment (hybrid Ag microstructures) left at the bottom of the falcon tube was extensively washed with Milli-Q water until a pH of ca. 6 was achieved. The hybrid particles were then redispersed in 2 mL of Milli-Q water. In the case of “activated” vermiculite, [13] the scaffold was treated with 40 mL of a 0.1 M solution of NaOH (1 h) after the initial cleaning cycles with NaCl solutions.

### 2.3. SERS Characterization 

The sample for SERS characterization was prepared as follows. Twenty microliters of resuspended hybrid Ag microstructures were diluted to 100 µL with Milli-Q water. Then, 3 µL of a 1 mM benzenethiol (BT) ethanolic solution was added. The mixture was left under gentle shaking for 1 h before acquiring the SERS spectra.

### 2.4. Instrumentation 

Scanning electron microscopy (SEM, JEOL JSM 6400, acceleration voltage of 20 kV, JEOL Ltd., Akishima, Tokyo, Japan) was used to analyze the elemental composition of the particles by energy-dispersive X-ray spectroscopy (EDS). Transmission electron microscopy (TEM, JEOL Ltd.company, Akishima, Tokyo, Japan) images were acquired by using a JEOL 1011 operating at 100 kV; TEM samples were prepared by drying the samples on carbon−Formvar-coated 200 mesh copper grids, Extinction spectra were obtained using a Thermo Scientific Evolution 201 UV-visible spectrophotometer (Thermo Fisher Scientific, Waltham, MA, US). SERS measurements were performed using a Renishaw InVia Reflex confocal microscope (Renishaw, Wotton-under-Edge, United Kingdom). equipped with high-resolution gratings (2,400 or 1,200 grooves per cm for the visible or NIR, respectively) with additional band-pass filter optics, a confocal microscope and a 2D-CCD camera. The lasers (three excitation laser lines = 514, 633 and 785 nm) were focused on the colloidal sample by employing a macro-lens of 30 mm focal length (0.17 NA). Laser powers at the sample were 514 nm = 2.6 mW, 633 nm = 4.8 mW, and 785 nm = 38.6 mW. Spectra were typically acquired with 10 s exposure time. 

## 3. Results and Discussion

Vermiculite flakes were extensively washed with NaCl aqueous solutions to improve cation exchange. This cleaning process facilitates the replacement of interlayer cations with Na^+^ ions, which have only electrostatic interaction with the siloxamic cavities of vermiculite; they have been reported to be efficiently exchanged with Ag^+^ ions [11]. When immersed into 0.1 M silver nitrate solution, vermiculite (Ve) acquires a dark coal colour, indicating the reduction of silver ions and, thus, the formation of Ag^0^-coated vermiculite (Ve@Ag). Residual silver ions were then eliminated by washing using Milli-Q water. In the case of “activated” vermiculite (AVe), after the initial cleaning step and before the immersion into AgNO_3_ solution, the scaffold was treated with a 0.1 M NaOH solution. Previous studies have shown that such chemical activation of vermiculite facilitates ion exchange, which, in turn, leads to an enhanced sorption capacity as compared to raw vermiculite [14,20].

Removal of the silicate network is achieved by dissolution with hydrofluoric acid (HF). Simultaneously, the HF treatment promotes the formation of a MgF_2_ skeleton, which acts as a support for the metallic silver. Overall, this process leads to the generation of hybrid Ag microstructures which are found as a precipitate at the bottom of the vial. It is worth noting that we also investigated the possibility of adding an external reducing agent (NaBH_4_) to the vermiculite/AgNO_3_ mixture prior to the washing steps. However, this primarily leads to the formation of large content of small colloidal nanoparticle in the bulk solution.

**Scheme 1:**
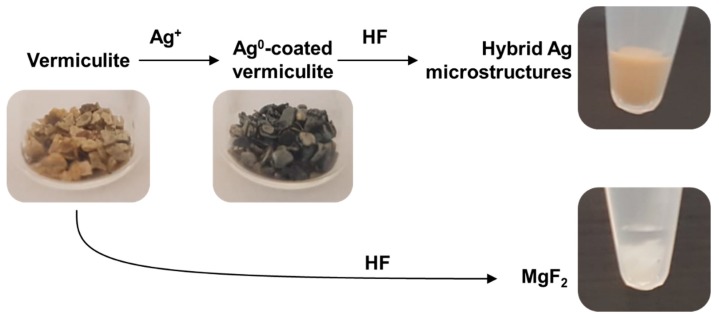
Outline of the synthesis of hybrid Ag microstructures. Optical images of the dried vermiculite flakes before and after immersion into AgNO_3_ solution are also included together with those of resuspended hybrid Ag materials and magnesium fluoride (MgF_2_) crystals upon hydrofluoric acid (HF) treatment.

Scheme 1 outlines the fabrication process and, for the sake of visual comparison, also shows optical photos of vermiculite flakes before and after the immersion into AgNO_3_ solution. We also report optical photos of resuspended hybrid Ag microstructures and vermiculite flakes upon HF treatment, which exhibit the characteristic brown-grey color of silver nanomaterials and the white pale color of MgF_2_ crystals, respectively.

**Figure 1:**
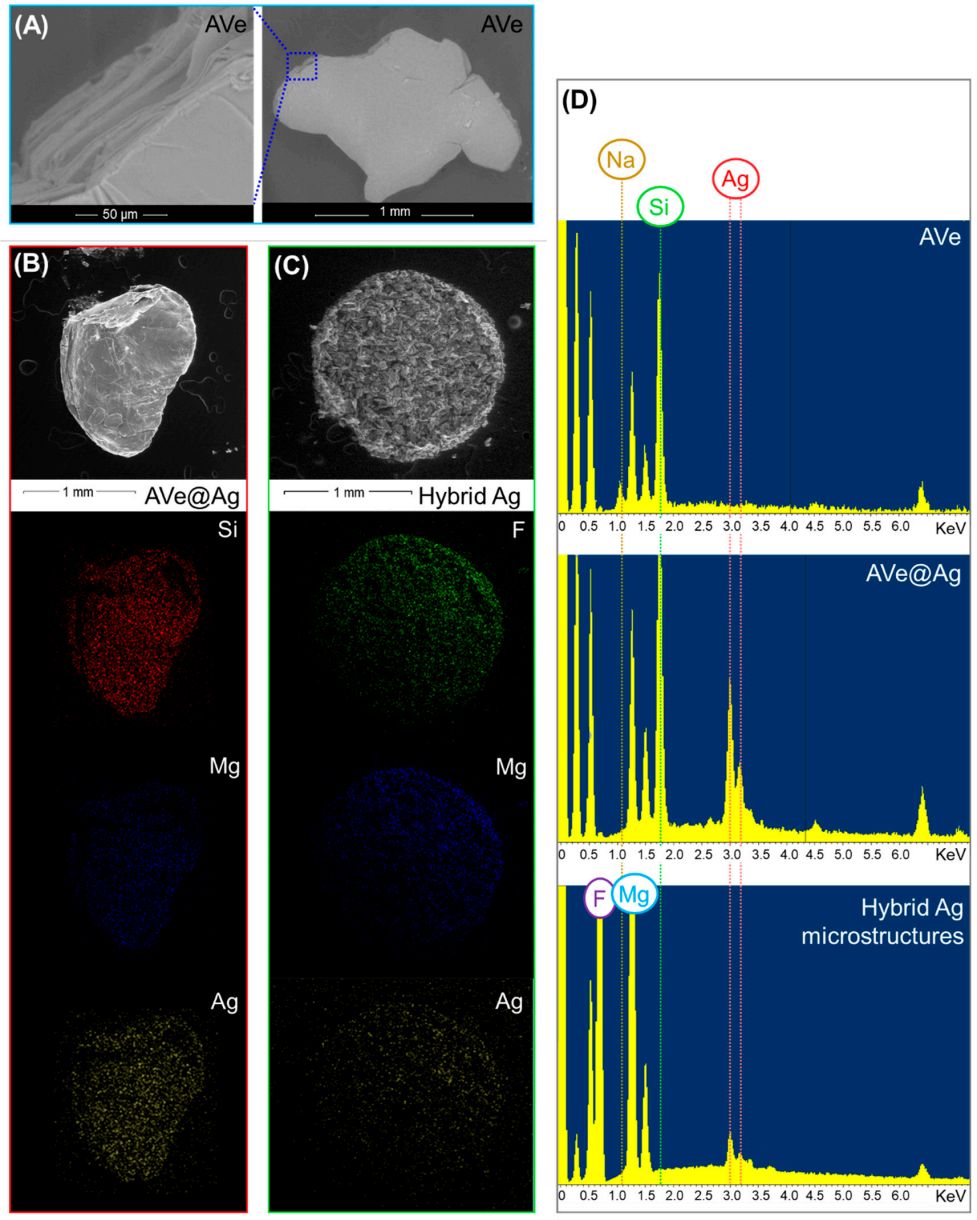
(**A**–**C**) Representative environmental scanning electron microscopy (ESEM) images of (**A**) activated vermiculite (AVe), (**B**) silver-coated activated vermiculite (AVe@Ag), and (**C**) hybrid Ag microstructures (from AVe). Elemental maps of (**B**,**C**) obtained via energy-dispersive X-ray (EDX) analysis are also shown. (**D**) EDX spectra of the illustrated structures.

**Figure 2:**
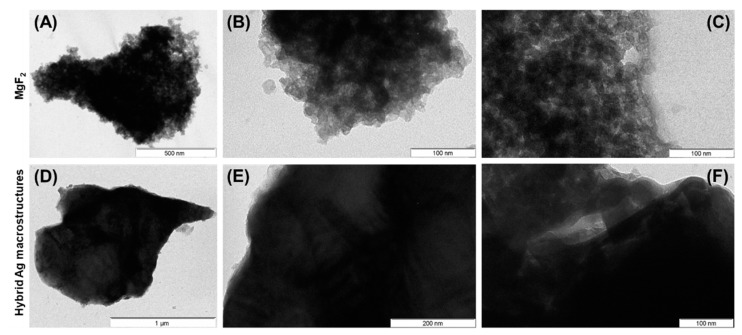
Representative transmission electron microscopy (TEM) images of (**A**–**C**) MgF_2_ from AVe and (**D**–**F**) hybrid Ag microstructures from AVe.

An ESEM–EDX characterization of individual particles from each step of the fabrication process is reported in Figure 1A–D and Figure S1. Layered AVe flakes (Figure 1A) are characterized by a large surface area available for Ag^+^ retention. As expected, silver deposition onto the vermiculite scaffold (AVe@Ag, Figure 2B) takes place, to a larger extent, via displacement of sodium counterions. This is corroborated by the disappearance of the Na feature from the EDX spectra (Figure 1D and Table S1), while the observed elemental ratios of Si vs. Mg, Fe, and Al (Table S1) do not qualitatively undergo critical alterations upon silver treatment. On the other hand, Si removal upon HF treatment is indicated by EDX analysis of hybrid Ag microstructures (Figure 1D).

Figure 2 compares representative TEM images of hybrid Ag microstructures from AVe and their MgF_2_ counterpart (Figure 2A–C vs. Figure 2D–F, respectively). A qualitative comparison shows an overall different structural pattern between the two samples, which is more clearly visible at the edges of the particles (Figure 2B,C vs. Figure 2E,F), thus providing an additional indicator that metallic silver is retained at the microparticle surfaces. 

**Figure 3:**
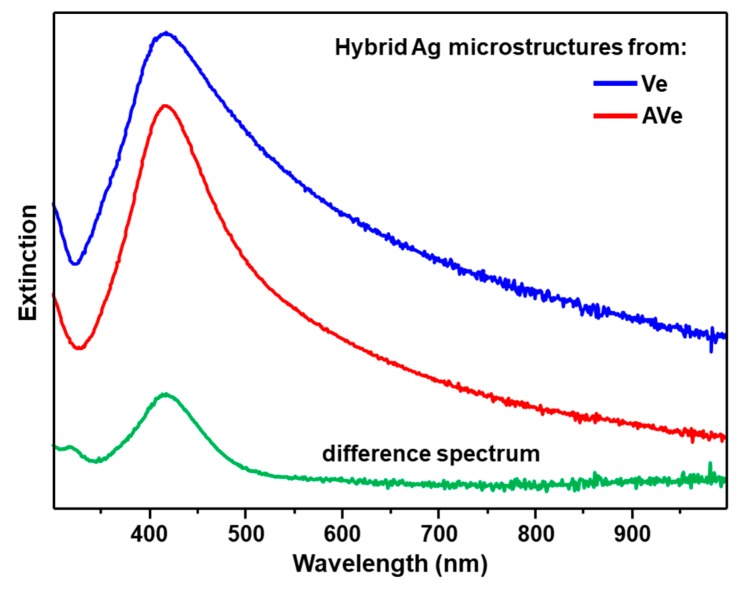
Extinction spectra of hybrid Ag microstructures from Ve (blue curve) and AVe (red curve). Green curve is the difference spectrum obtained by subtracting the blue spectrum from the red one.

Electron microscopy studies, as well as the EDX analysis of individual hybrid Ag microstructures, did not provide conclusive evidence about potential differences between Ve or AVe derived materials. Thus, we employed highly averaged optical characterization approaches to unravel the potential impact of the chemical activation of vermiculite on the silver coating. Firstly, hybrid Ag microstructures resuspended in water were interrogated via extinction spectroscopy. The presence of nanostructured Ag features is confirmed by an intense and broad localized surface plasmon resonance (LSPR) band centered at ca. 420 nm, which emerges from an extended scattering signal background (Figure 3). The comparison of the extinction spectra also appears to reveal a more pronounced LSPR contribution, as compared to the scattering background, for hybrid materials obtained starting from AVe scaffolds. This is qualitatively highlighted in Figure 3 by digital subtraction of the blue curve (extinction spectrum of hybrid Ag microstructures from Ve) from the red curve (extinction spectrum of hybrid Ag microstructures from AVe), resulting in a difference spectrum (green curve) that displays a LSPR band centered at ca 420 nm. 

**Figure 4:**
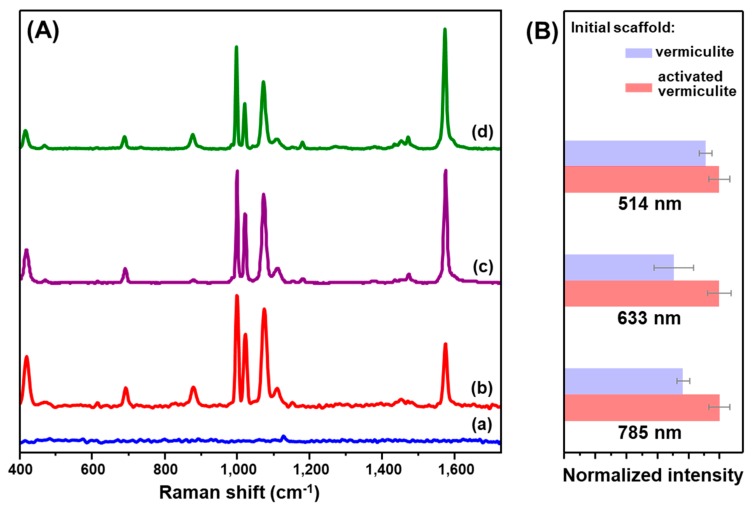
(**A**) Surface-enhanced Raman spectroscopy (SERS) spectra of (a) hybrid Ag microstructures from AVe and (b, c, d) benzenethiol (BT) on hybrid Ag microstructures from AVe by exciting with (b) 785 nm, (c) 633 nm, and (d) 514 nm lasers. (**B**) Normalized SERS intensity of the BT 999 cm^−1^ band (peak height) for hybrid Ag microstructures from Ve and AVe at different excitation wavelengths (error bars for N = 3). SERS spectra were collected using a lens for macrosampling.

Such a dissimilarity in the overall plasmonic response is mirrored by the results obtained from the surface-enhanced Raman scattering (SERS) characterization of the hybrid Ag microstructures. SERS response of the materials has been tested at three excitation wavelengths (514, 633, and 785 nm) using benzenethiol (BT) as a molecular probe. Figure 4A shows the SERS spectra of the resuspended hybrid Ag microstructures before and after the addition of the probe, which are characterized by the appearance of the characteristic set of intense features from BT on silver [12]. We can attribute the origin of the SERS signal mostly to the BT molecules adsorbed at the nanostructured metallic features where the largest local field enhancements are localized. A qualitative comparison of the SERS efficiency of the hybrid Ag microstructures from the two different scaffolds (Ve vs. AVe) is shown in Figure 4B, where the intensity of the narrow 999 cm^−1^ band, ascribed to the ring breathing [21], is reported for the different excitation wavelengths. The results suggest that chemical modification of the vermiculite appears to consistently improve the SERS response of the hybrid Ag microstructures for all investigated lasers. This may derive from the larger content of nanostructured Ag features, as suggested in Figure 3. The properties of hybrid Ag microstructures make them suitable to be used as stable microscopic carriers of nanostructured features, which can be straightforwardly integrated as analyte accumulators in suspension. We tested this possibility by dispersing a low amount of hybrid Ag microstructures into BT solutions at different concentrations. The plasmonic microstructures were then easily recovered by quick sedimentation and redispersed into a small volume prior to the SERS measurements. The BT marker bands were detected down to the pM level of the molecular probe (Figure S2).

In summary, we have reported the use of vermiculite as a low-cost template for the straightforward and easy scalable preparation of complex 3D silver hybrid materials in suspension. The results indicate that silver ions efficiently exchange with Na^+^ ions at the outer silicate surfaces and undergo reduction to Ag^0^. Removal of the silicate network by HF treatment generates a MgF_2_ skeleton decorated with metallic silver. SERS characterization displayed a high enhancing efficiency across a broad range of excitation wavelengths, while the additional stability and ease of separation enabled the application of hybrid Ag microstructures as accumulators for SERS detection in suspension. Main advantages of this new class of potential SERS substrates are the (i) the very low-cost and availability of vermiculites as scaffolds; (ii) the extreme simplicity of the fabrication protocol; (iii) the outstanding scalability, as grams of hybrid Ag microstructures can be produced at once; and (iv) the high transmittance of the supporting backbone (MgF_2_) in the spectral range of interest for SERS.

## Supplementary Materials

The following are available online at https://www.mdpi.com/2079-4991/10/3/481/s1, Figure S1: ESEM-EDX characterization of vermiculites, silver-coated vermiculites particles and hybrid silver microstructures. Table S1: Atomic ratios obtained from the ESEM-EDX characterization. Figure S2: SERS detection of benzenethiol with hybrid silver microstructures.
