# Supplementary material for: Fabrication of Hybrid Silver Microstructures from Vermiculite Templates as SERS Substrates
Source: Nanomaterials (Basel). 2020 Mar 7;10(3):481. doi: 10.3390/nano10030481 (PMC7153242; doi:10.3390/nano10030481)
Supplement: Supplementary file 1 [file nanomaterials-10-00481-s001.pdf]

Supplementary Materials

Fabrication of hybrid silver microstructures from vermiculite templates as SERS substrates

Nicolas Pazos-Perez,[1] Luca Guerrini [1,*], and Ramon A. Alvarez-Puebla [1,2,*]

[1] Department of Physical and Inorganic Chemistry and EMaS, Universitat Rovira I Virgili, Carrer de Marcel·lí Domingo s/n, Edifici N5, 43007 Tarragona, Spain.; nicolas.pazos@urv.cat (N.P.P.)

[2] Institució Catalana de Recerca i Estudis Avançats (ICREA), Passeig Lluís Companys 23, 08010 Barcelona, Spain.

[*]Correspondence: luca.guerrini@urv.cat (L.G.); ramon.alvarez@urv.cat (R.A.A.P.)

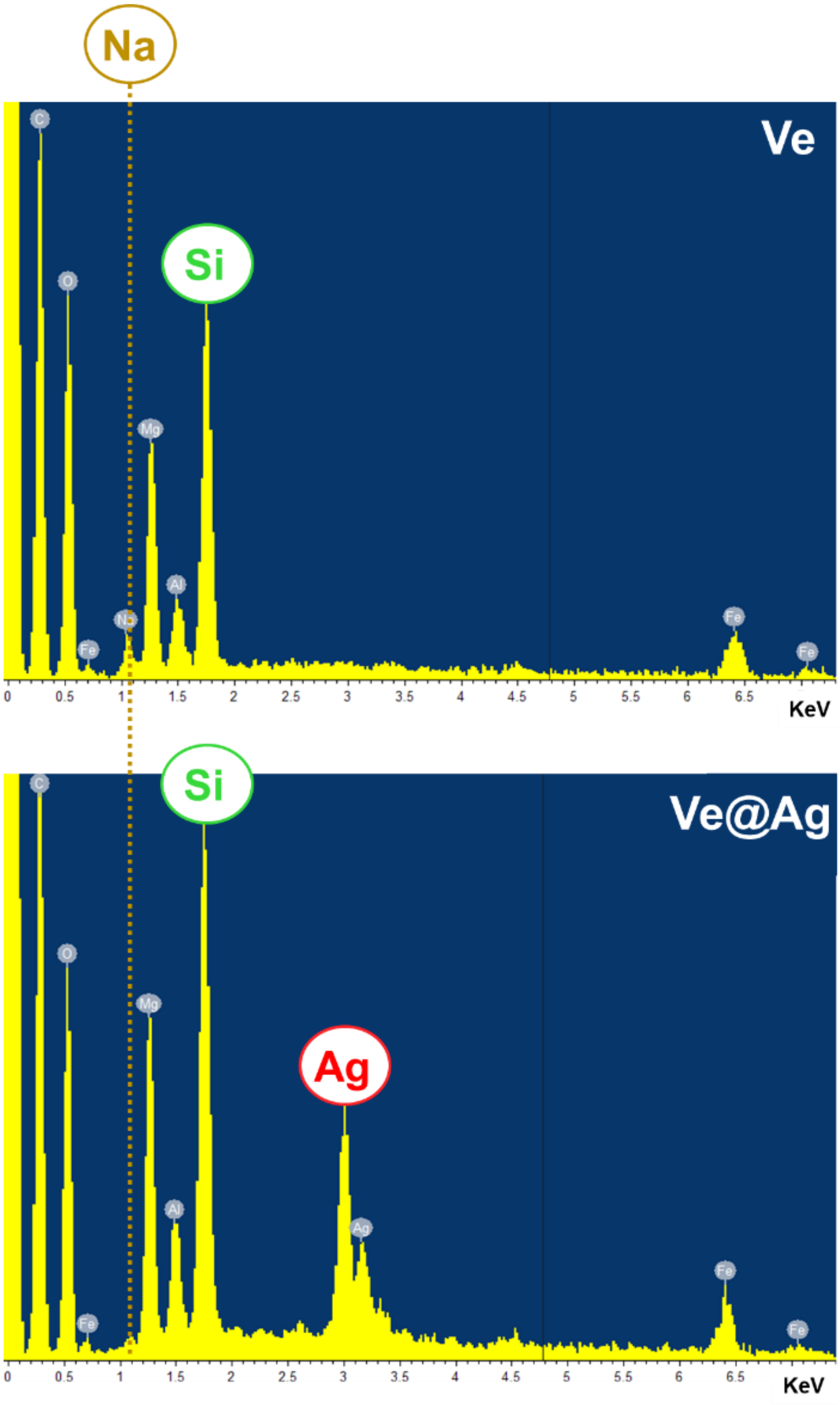

Figure S1. Energy-dispersive X-ray (EDX) spectra of vermiculite (Ve) and silver-coated vermiculites (Ve@Ag).

**Sample**	**Ag/Si**	**Mg/Si**	**Al/Si**	**Fe/Si**	**O/Si**	**Na/Si**
**Vermiculite (Ve)**	0	0.75	0.16	0.20	4.18	0.16
**Ag-coated vermiculite (Ve@Ag)**	0.31	0.78	0.22	0.19	4.19	0
**Activated vermiculite (AVe)**	0	0.68	0.24	0.19	4.17	0.17
**Ag-coated activated vermiculite (AVe@Ag)**	0.28	0.71	0.25	0.22	4.05	0

Table S1. Atomic ratios obtained from the ESEM-EDX characterization illustrated in Fig. 1 and S1.

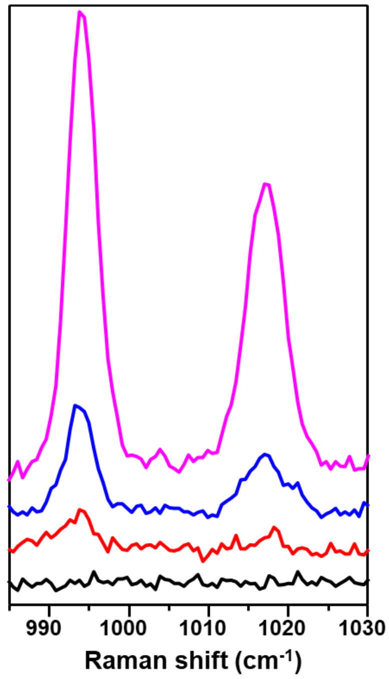

Figure S2. SERS detection of benzenethiol. Detail of the SERS spectra of hybrid Ag microstructures from activated vermiculite in the presence of decreasing concentration of BT, from the top to the bottom: 10 nM, 1 nM, 10 pM, 1 pM. SERS experiments were performed as follows. 5 µL of the Ag microstructures suspension were dispersed with 995 µL of BT aqueous solutions at decreasing concentrations. The samples were left under shaking for 2 hours, then the Ag microstructures were left to sediment. 980 µL of the supernatant were removed, and the microstructures were redispersed in suspension prior to the SERS analysis. SERS spectra were acquired with 20 seconds exposure time, 5 accumulations and by illuminating with a 633 nm laser.
